# Steroid Signaling within Drosophila Ovarian Epithelial Cells Sex-Specifically Modulates Early Germ Cell Development and Meiotic Entry

**DOI:** 10.1371/journal.pone.0046109

**Published:** 2012-10-02

**Authors:** Lucy X. Morris, Allan C. Spradling

**Affiliations:** Howard Hughes Medical Institute, Department of Embryology, Carnegie Institution, Baltimore, Maryland, United States of America; Technische Universität Dresden, Germany

## Abstract

Drosophila adult females but not males contain high levels of the steroid hormone ecdysone, however, the roles played by steroid signaling during Drosophila gametogenesis remain poorly understood. Drosophila germ cells in both sexes initially follow a similar pathway. After germline stem cells are established, their daughters form interconnected cysts surrounded by somatic escort (female) or cyst (male) cells and enter meiosis. Subsequently, female cysts acquire a new covering of somatic cells to form follicles. Knocking down expression of the heterodimeric ecdysteroid receptor (EcR/Usp) or the E75 early response gene in escort cells disrupts 16-cell cyst production, meiotic entry and follicle formation. Escort cells lose their squamous morphology and unsheath germ cells. By contrast, disrupting ecdysone signaling in males does not perturb cyst development or ensheathment. Thus, sex-specific steroid signaling is essential for female germ cell development at the time male and female pathways diverge. Our results suggest that steroid signaling plays an important sex-specific role in early germ cell development in Drosophila, a strategy that may be conserved in mammals.

## Introduction

Pulses of the Drosophila steroid hormone ecdysone coordinate the major transitions that occur during development and growth, as well as adult nutritional and circadian cycles [1], [2], [3]. Ecdysone pulses are initiated by cues from insulin, nitric oxide, TGFβ and other signals, and activate a well-characterized pathway in target cells involving the heterodimeric receptor EcR/Usp and the downstream genes *E75, DHR3, ftz-f1, Hr39* and others (reviewed in [4]).

**Figure 1:**
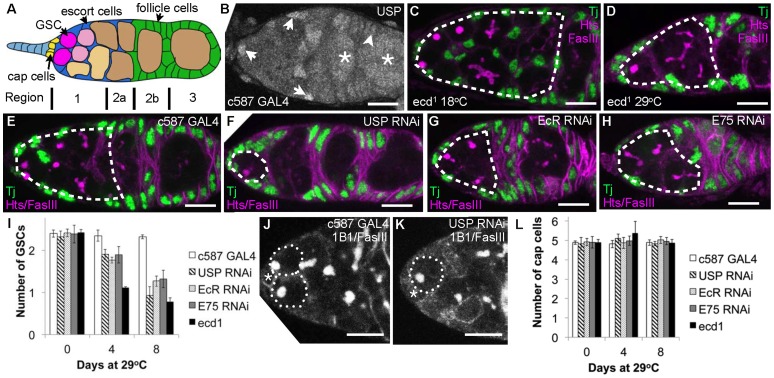
Ecdysone signaling maintains germarial size and GSC number. A) Diagram of the Drosophila germarium: terminal filament cells (light blue), cap cells (yellow), GSCs (magenta), cystoblasts (pink), cysts (beige), escort cells (dark blue), follicle cells (green). Subregions 1–3 of the germarium are indicated. B) anti-USP staining of somatic escort cell nuclei (arrows), follicle cell nuclei (arrowhead) and germ cells within forming follicles (asterisks). C–H) Regions 1 and 2a of the germaria containing GSCs and cysts (dashed outline) appear smaller when ecdysteroid signaling is reduced. C) *ecd^1^* 18^o^C control; D) *ecd^1^* 29^o^C day 4; E) c587 alone 29^o^C day 8; F) c587::USP RNAi 29^o^C day 8; G) c587::EcR RNAi 29^o^C day 8; H) c587::E75 RNAi 29^o^C day 8. Green, somatic cells (anti-Tj), magenta, cell membranes and spectrosome/fusome (anti-Hts and anti-FasIII). I) Time course showing the number of GSCs present in germaria from controls and flies in which ecdysone signaling was reduced for the duration shown as described. J-K) Control germaria usual have two to three GSCs (J, dashed outline), whereas c587:USP RNAi flies after 8 days at 29°C usually have only one (K, dashed outline), white: cell membranes and spectrosome/fusome (anti-Hts and anti-FasIII). Asterisk marks the position of the cap cells. L) The number of cap cells was counted in germaria from controls and flies in which ecdysone signaling was reduced for the duration shown. Cap cell number is not affected by reducing ecdysteroid signaling. Error bars indicate s.d. Scale bar: 10 µm.

Oogenesis involves many developmental transitions and exquisitely balanced responses to changing environmental conditions, at least some of which are regulated by ecdysone. Oogenesis in adult Drosophila is maintained by two to three germline stem cells (GSCs), located at the anterior of each string of developing egg chambers within a structure called a germarium (Fig. 1A). Somatic cap cells produce signals that hold GSCs within the niche environment and prevent differentiation (see Fig. 1A; reviewed in [5]). Altered steroid signal reception in GSCs affects their stability, responsiveness to niche signals, and their daughter’s ability to promptly initiate development [6], [7]. Niche associated somatic escort cells are likely involved, because these cells were altered in shape and adhesivity when signaling to GSCs was disrupted [6]. One likely function of ecdysone is to help coordinate GSC activity with the nutritional levels as sensed by insulin production [8].

Formation of a mature egg from a GSC requires oocytes to undergo a developmental progression involving 14 recognized stages (stages 1–14). GSC daughters (cystoblasts, CBs) undergo four synchronous divisions progressively forming 2-, 4-, 8- and finally 16-cell germline cysts. During cyst formation cytokinesis is incomplete, leaving the cells within the cyst connected via ring canals. Gamete sex (e.g. sperm or egg) is determined within cysts and meiosis is initiated by the time the 16-cell cyst stage is reached. The continual presence of somatic escort cells, which completely wrap both GSCs and cysts, is required for cyst differentiation. After meiosis is initiated, cysts shed their escort cell covering and become surrounded by somatic follicle cells giving rise eventually to a new ovarian follicle, which then buds off from the germarium (Fig. 1A). Whether steroid hormone signaling has a role during these early events of Drosophila oogenesis is unknown.

A second period under the control of steroid signals occurs near the end of oogenesis: Ecdysone regulates the transition of follicles though a checkpoint at stage 8 that prevents the onset of vitellogenesis and egg maturation if nutritional resources are insufficient [9], [10], [11]. Additionally, once past the checkpoint, ecdysone-mediated signaling in somatic follicle cells helps orchestrate egg completion including eggshell morphogenesis [12], [13], [14].

Steroid signaling also plays a role at multiple stages of mammalian oogenesis, including gamete sex determination (reviewed in [15]). Although steroid signaling previously had no known role in Drosophila sex determination (reviewed in [16]), ecdysone does play a sex differential role during adulthood. Ecdysone signaling pathway genes are differentially expressed between ovaries and testes and are functionally required for female but not for male fertility [10], [17], [18].

As steroid hormone signaling is a key regulator of developmental transitions we investigated whether ecdysone controls events in early Drosophila oogenesis. We show that ecdysteroid signaling is important for several steps of early female gametogenesis downstream from the GSC including 16-cell cyst formation, meiotic entry, and follicle formation. Steroid signaling acts in the somatic cells enveloping germline cysts in females but not in structurally similar male somatic cells. Gametogenesis diverges in the two sexes during cyst formation. For example, male meiosis lacks recombination and requires male-specific cell cycle genes (reviewed in [19], [20]). Our results argue that ecdysone-mediated signaling represents an early branch point between male and female germline development. Thus, in Drosophila, as well as mammals, sexually dimorphic steroid hormone signaling acts at the time development diverges between male and female germ cells.

## Results

### Early Oogenesis Requires Nuclear Hormone Receptor Function within Somatic Cells

To investigate whether ecdysone signaling is required for early oogenesis, we reduced whole fly hormone levels using a temperature sensitive *ecdysoneless* mutant (*ecd^1^*). *ecd* mutant flies were maintained at 18°C to provide essential signaling during development then moved to the restrictive temperature of 29°C, which reduces circulating ecdysone to 30% of wild-type levels [17]. Additionally, we used RNAi to knock down expression of ecdysone receptor genes (*ultraspiracle, usp* and the *ecdysone receptor*, *EcR*) or the early ecdysone effector gene (*E75*). To identify the ecdysone-responsive cell population, ecdysone-signaling activity was reduced in a limited group of cells. Knock down was confined to escort and undifferentiated follicle cells by driving RNAi expression using a GAL4 driver line (c587) expressed in these somatic cell types but not in germ cells (Fig. S1A–B). RNAi expression was prevented during development using a temperature sensitive gal80 repressor and by maintaining flies at 20^o^C. When adult animals were switched to 29°C for 8 days, RNAi-mediated gene knock down occurred in the somatic cells of the germarium. For example, the Usp co-receptor is expressed in both somatic and germ cells of the germarium (Fig. 1B). RNAi directed against Usp driven by the c587 GAL4 line eliminated Usp antibody staining specifically in somatic cells, but not germ cells (Fig. S1C, D).

**Figure 2:**
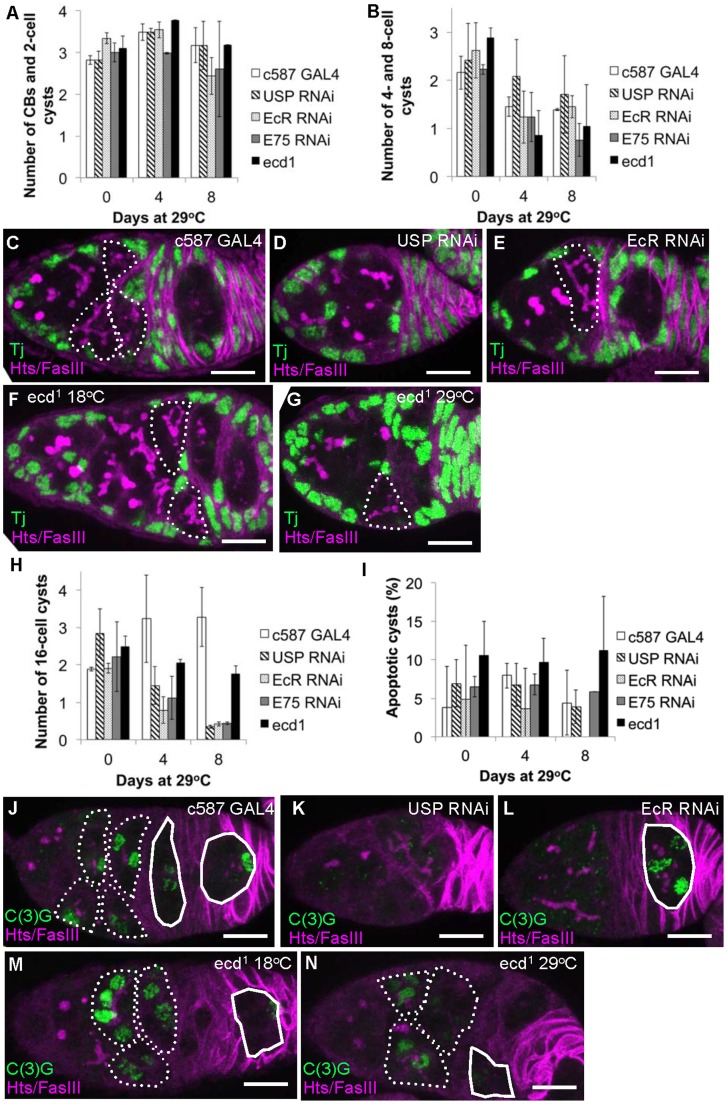
Ecdysone signaling is needed to efficiently form 16-cell cysts and enter meiosis. A–H) CB and cyst number in control animals and animals with compromised ecdysone signaling. A, B) CB and 2-, 4- and 8-cell cyst number are unaffected whereas the number of 16-cell cysts is reduced (C–H). C–G) 16-cell cysts outlined, no outline indicates an absence of 16-cell cysts. C) c587 alone 29^o^C day 8; D) c587::USP RNAi 29^o^C day 8; E) c587::EcR RNAi 29^o^C day 8; F) *ecd^1^* 18^o^C control; G) *ecd^1^* 29°C day 4. Green: somatic cells (anti-Tj), magenta: cell membranes and spectrosome/fusome (anti-Hts and anti-FasIII). I) No change in TUNEL positive 16-cell cyst number was seen when ecdysteroid signaling was limited. J-N) Germ cells and cysts within germaria from control flies and flies where ecdysone signaling was compromised were stained for C(3)G protein to reveal synaptonemal complex-containing cells. Region 2a cysts (dashed outline), region 2b follicles (solid outline) and region 3 follicle (solid outline) indicated. No outline indicates absence of C(3)G positive cysts or follicles. Green, synaptonemal complex (anti-C(3)G), magenta, cell membranes and spectrosome/fusome (anti-Hts and anti-FasIII). J) c587 alone 29^o^C day 8; K) c587::USP RNAi 29^o^C day 8; L) c587::EcR RNAi 29^o^C day 8; M) *ecd^1^* 18^o^C control; N) *ecd^1^* 29^o^C day 4. Scale bar: 10 µm. Error bars indicate s.d.

The effects on early oogenesis of reducing ecdysone synthesis or knocking down pathway receptors were dramatic. The regions of germaria containing GSCs and cysts (regions 1 and 2a, see Fig. 1A) appeared significantly smaller (Fig. 1C–H). We explored two possible explanations for this reduction in germarium size. Firstly, loss of GSCs could decrease the rate of cyst production or secondly, blocked cyst differentiation might lead to the absence of larger, mature cysts.

**Figure 3:**
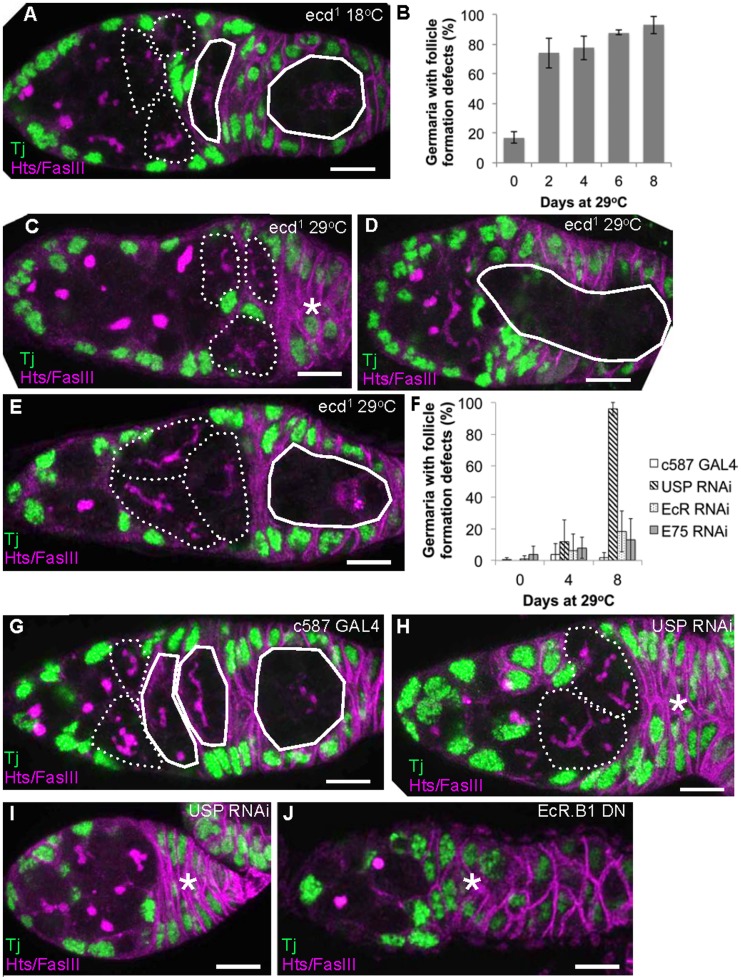
Follicle formation requires ecdysone signaling. A–J) Germaria from control flies and animals in which ecdysone signaling was compromised following temperature shift for the indicated periods, were analyzed to determine the number of region 2a (dashed outline), region 2b (solid outline) and region 3 (solid outline) 16-cell cysts. Asterisk  =  missing follicle(s). Germaria from *ecd^1^* animals (B) and animals in which ecdysone signaling components were knocked-down (F) were scored as to whether follicle formation was defective by analysing the number of cysts present (at least one cyst is normally present in each of regions 2a, 2b and 3) and whether the location and morphology of these cysts appeared normal. A) *ecd^1^* 18°C control; C–E) *ecd^1^* 29°C day 4; G) c587 29^o^C day 8; H–I) c587::USP RNAi 29C day 8; J) c587::EcR.B1 dominant °negative 29°C day 8. Green: somatic cells (anti-Tj), magenta: cell membranes and fusome (anti-hts and anti-FasIII). Error bars indicate s.d. Scale bar: 10 µm.

To establish whether GSCs were maintained and the normal developmental sequence of developing germline cysts occurred when ecdysone signaling was reduced, germaria were dissected and stained with an antibody directed against Hu-li tai shao (Hts) [21]. Hts is located within an endoplasmic reticulum-like structure present in germ cells called a spectrosome in GSCs and CBs and a fusome in cysts [22]. As the spectrosome/fusome branches each time a CB or cyst division occurs the number of branches in a fusome can be used to establish cyst size and number. Additionally, the spectrosome within GSCs is positioned adjacent to the cap cells enabling GSC number to be determined.

**Figure 4:**
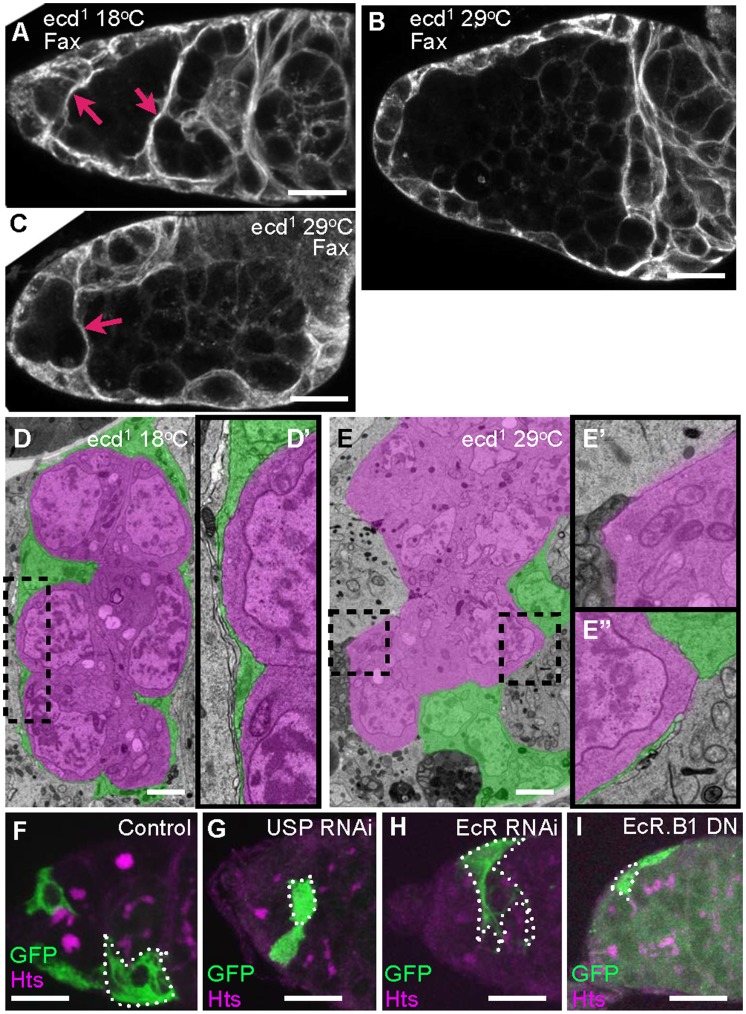
Somatic cells change shape when ecdysone signaling is reduced. A–C) Escort and early follicle cell processes (labeled with anti-Fax) entirely surround each germline cyst and early follicle in control germaria (A, red arrows) but are completely (B) or partially (C, red arrow indicates intact process) retracted when *ecd^1^* flies are shifted to 29^o^C. A) *ecd^1^* 18^o^C control; B/C) *ecd^1^* 29^o^C day 8. Scale bar: 10 µm. D–E”) EM analysis of somatic process retraction, germ cells in a single cyst pseudocoloured magenta and escort cells green. D′ is an enlargement of outlined region in D and E′ and E′′ are enlargements of outlined region within E. D/D′) *ecd^1^* 18^o^C control; E/E′/E′′) *ecd^1^* 29^o^C day 4 Scale bar: 2 µm. G) Knock down of *usp* in a sub-population of escort cells (green, single cell outlined) causes cell shape changes (compare to control, F). H) Knock down of *EcR* expression in a single escort cell (outlined) does not change the cell shape. I) Over expression of EcR.B1 dominant negative in a sub-population of escort cells (green, single cell outlined) does cause shape changes. Green: GFP and RNAi expression, magenta: cell membranes and fusome (anti-Hts). F) Flipout::GFP 29° day 7; G) Flipout::GFP USP RNAi 29°C day 7; H) Flipout::GFP EcR RNAi 29°C day 7; I) Flipout:: EcR.B1 dominant negative 29°C day 5. Scale bar: 10 µm.

### Ecdysteroids Maintain Germline Stem Cell Number

Using spectrosome position, GSC number was determined in *ecd* mutants and animals in which ecdysone signaling pathway components had been knocked down. Whereas controls always displayed two or three GSCs, germaria with compromised steroid hormone signaling frequently only had one (Fig. 1I–K). Loss of GSCs was particularly rapid in *ecd^1^* females, which lost half of their GSCs within four days of a shift to the restrictive temperature (Fig. 1I).

**Figure 5:**
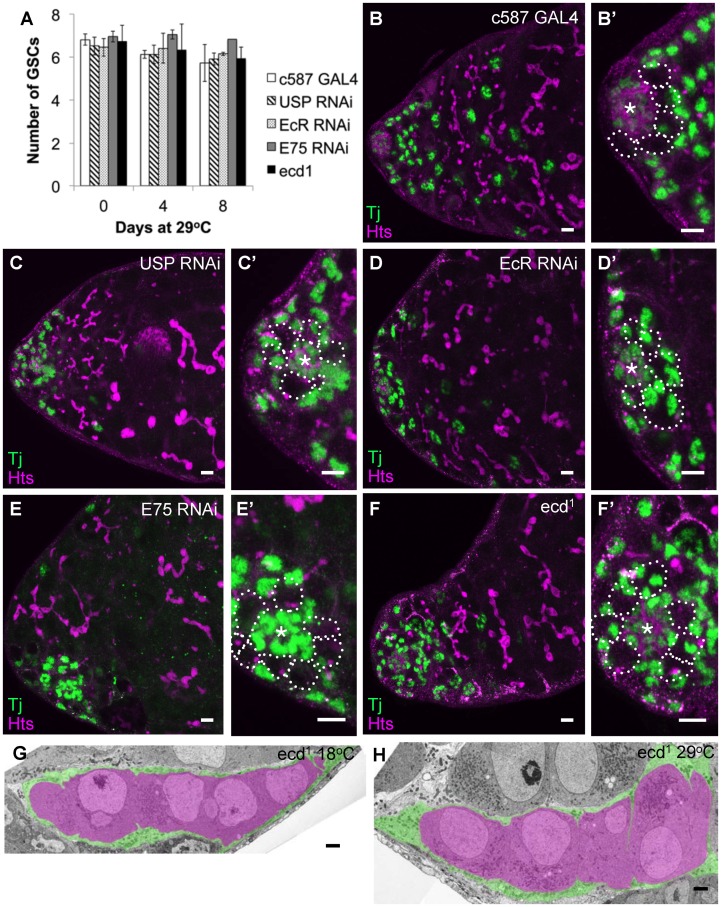
Ecdysone signaling is not required for GSC maintenance, early germ cell development, or somatic cell shape in the testis. A) The number of male GSCs was counted after the indicated periods following a shift to 29°C to compromise ecdysone signaling as indicated. Error bars indicate s.d. B) c587 alone 29°C day 8; C) c587::USP RNAi 29°C day 8; D) c587::EcR RNAi 29°C day 8; E) c587::E75 RNAi 29°C day 8; F) *ecd^1^* 29°C day 4. (B′–F′: enlarged regions). GSCs outlined in B′, C′, D′, E′ and F′ and asterisk position of hub cells. Green: somatic cells (anti-Tj) magenta: cell membranes and fusome (anti-hts and anti-FasIII). Scale bar: 10 µm. G, H) EM analysis of *ecd^1^* cysts from males kept G) at 18°C, or H) 29°C day 8. Magenta: pseudocolour (germ cells within a single cyst), green: pseudocolour (cyst cells in contact with the pseudocoloured cyst). Scale bar: 2 µm.

Cap cells are a critical component of the GSC niche, and expansion and reduction of this cell population can dictate GSC number [23], [24], [25]. We therefore tested whether the reduction in GSC number observed when ecdysteroid signaling is limiting is caused by a reduction in cap cell number. However, both control germaria and germaria with reduced hormone signaling contained four or five cap cells, indicating that GSC loss in these experiments is not caused by cap cell loss (Fig. 1L).

### Ecdysone Mediated Signaling in Somatic Cells is Required to Form Meiotic 16-cell Cysts

Compromised ecdysone signaling might also reduce the size and germ cell content of germaria by affecting cyst formation or stability. Normally, CBs undergo a series of four synchronous cell cycles to generate 2-, 4-, 8- and 16-cell cysts. We recorded the number and stage of cysts in wild type, *ecd* mutant and ecdysone signaling depleted germaria (Fig. 2A, B, H). Wild-type germaria contain one to two CBs and 2-cell cysts, one 4- and one 8-cell cyst and one to two 16-cell cysts. Down regulation of somatic cell ecdysteroid signaling had little effect on CB, 2-, 4- and 8-cell cyst number (Fig. 2A, B) but dramatically lowered the number of 16-cell cysts (Fig. 2C–H, 16-cell cysts outlined in C–G). Surprisingly, the number of 16-cell cysts in *ecd^1^* animals kept at the restrictive temperature barely changed, but rather than indicating a weaker effect on cyst formation, this is probably because pre-existing 16-cell cysts present at the moment of temperature shift were unable to bud off as new follicles (see below).

Under conditions of limiting nutrition, both stage 8 follicles and 16-cell cysts in region 2a undergo apoptosis to preserve nutritional resources [8]. Ecdysone levels control entry into apoptosis by stage 8 follicles [9]. To establish whether ecdysteroid-dependent entry into apoptosis could similarly explain the lack of 16-cell cysts in germaria with reduced ecdysteroid signaling, apoptotic cysts were counted. However, no increase in apoptotic cyst number was observed (Fig. 2I), suggesting that ecdysteroid signaling regulates the formation, not the survival of 16-cell cysts.

One of the most fundamental events of early oogenesis is entry into meiosis, a process that commences in newly formed 16-cell cysts. The signals triggering entry into meiosis remain poorly understood, especially in metazoans, but in yeast involve nutrient limitation (reviewed in [26]). Because the onset of meiosis (pre-meiotic S phase) occurs shortly after 16-cell cyst formation, we investigated whether ecdysone signaling affects meiotic induction by immunostaining for C(3)G, a component of the synaptonemal complex [27]. In controls, two to three C(3)G positive 16-cell cysts were found in each germarium, as expected (Fig. 2J and M dashed line outlines region 2a cysts and solid line region 2b or 3 follicles). However, the reduction in 16-cell cyst production caused by knock down of ecdysone signaling components resulted in an absence of C(3)G staining in region 2a/b of many germaria (Fig. 2K–L). A few older cysts that probably entered meiosis before RNAi became effective stained positively (Fig. 2L, solid line). 16-cell cysts present in *ecd* mutant germaria, which likely formed before the temperature shift and were unable to continue oogenesis due to follicle formation defects (see below) stained positively for C(3)G (Fig. 2N, dashed line). Thus, compromised ecdysone pathway function severely impairs new 16-cell cyst formation and entry into meiosis.

### Follicle Formation Requires Steroid Hormone Signaling

After 16-cell cysts progress through meiosis I in region 2a of the germarium, their covering of escort cells is replaced by follicle cells that proliferate and eventually coat each new follicle. The apparent retention of already formed 16-cell cysts in *ecd^1^* germaria following temperature shift suggests that follicle formation might also require ecdysone signaling. Control germaria contained at least one ‘lens-shaped’ region 2b follicle and one spherical, region 3 follicle (Fig. 3A, solid lines). However, 70% of *ecd^1^* germaria already showed morphological defects in region 2b and/or 3 follicles within two days at the restrictive temperature (Fig. 3B). Defects included the absence of a region 2b or a region 3 follicle (Fig. 3C, position of missing 2b and 3 follicle marked by an asterisk) and/or fusions between adjacent late follicles (Fig. 3D, outlined). Additionally, the normal perpendicular alignment of 16-cell cysts relative to the A/P axis in region 2a (Fig. 3A, dashed lines), was often disrupted (Fig. 4E, dashed lines), which may impede follicle formation. These data provide evidence that follicle formation requires ecdysone signaling.

Unlike in *ecd* mutants, knock down of ecdysone signaling pathway components does not lead to the retention of 16-cell cysts due to a rapid block in follicle formation after temperature shift (Fig. 2H, Fig. 3B). However, follicle formation defects could occur at later time points after the pre-formed 16-cell cysts have formed follicles. Examination of germaria in which USP had been knocked down showed this to be the case. After 4 days at 29^o^C few germaria contained abnormal or missing region 2b or 3 follicles, but by day 8 defects were seen in 96±18% of germaria (Fig. 3F). Either no follicles of these stages were present (Fig. 3H, I, asterisk marks missing region 2b and 3 follicles), or the 2a cysts were abnormally shaped and oriented (Fig. 3I, compare with 3G). By contrast, follicle formation defects occurred less frequently when EcR or E75 were knocked down (Fig. 3F), possibly due to weaker gene knockdown. Follicle formation defects were also seen when ecdysteroid signaling was disrupted by the expression of a dominant negative EcR construct, which expresses a competitive inhibitor to the endogenous EcR [28] (Fig. 3J, asterisk marks position of missing 2b and 3 follicles, by day 8 at 29^o^C follicle formation defect were apparent in 5% (10/190) of control germaria and 47% (76/163) c587::UAS-EcR.DN germaria). Thus, canonical ecdysteroid signaling in somatic cells is important for follicle formation, in addition to its earlier roles. The difference in the penetrance of follicle formation defects seen between the *ecd* mutants and the ecdysone pathway member knock downs is likely due to the relatively slow kinetics and limited magnitude of gene knock down.

### Steroid Hormones Influence Somatic Cell Shape

Both escort cells and follicle cells interact dynamically with adjacent germ cells [29]. In region 1 and 2a, squamous escort cells wrap around each cyst, while in region 2b, newly generated follicle cells migrate and envelope germ cells as they are released by escort cells [30]. When steroid signaling was disrupted we noticed that the behavior of both types of somatic cell was altered. Dramatically, wrapping of cysts by escort cell membranes and region 2b follicles by follicle cell membranes was altered or abolished when ecdysone synthesis was reduced, as determined by both immunohistochemistry using anti-fax, which strongly labels escort and follicle cell membranes (Red arrows in Fig. 4A–C) and electron microscopy (Pseudocoloured green in Fig. 4D–E). Knocking down Usp in a subset of escort cells showed that unwrapping was an autonomous effect (Fig. 4F–G). Whereas control cells maintained thin microtubule rich membrane extensions that surrounded adjacent germ cells (Fig. 4F, single escort cell outlined), reducing *usp* expression caused normally squamous escort cells to resemble cuboidal epithelial cells (Figure 4G, single escort cell outlined). Similar somatic cell shape changes were not observed when EcR expression was knocked down (Fig. 5H, single escort cell outlined), but were seen when EcR.B1 dominant negative was over expressed (Fig. 5I, single escort cell outlined). Thus, the effectiveness of constructs in producing shape changes correlates with their ability to cause follicle formation defects.

### Ecdysteroids do not Influence Male Germ Cell Development

The early stages of germ cell development are largely conserved between male and female Drosophila. Both males and females maintain GSCs using JAK-STAT and BMP signals produced by niche cells and grow new 16-cell cysts within a covering of squamous somatic cells (escort cells of the ovary, cyst cells of the testis) prior to meiotic entry (reviewed in [5]). To investigate the role of ecdysteroids during early male germ cell development, we analyzed *ecd^1^* males and knocked down gene expression with c587-GAL4 driver, which is strongly expressed in somatic cyst progenitor cells and cyst cells of the testis and should exert a similar degree of pathway knock down in the testis as it did in the ovary (see Fig. S2). Male GSC number can be accurately determined by counting the number of spectrosome containing germ cells in direct contact with the hub cells, a distinctive cluster of somatic cells that generate the GSC niche. Provocatively, loss of male GSCs was not seen in *ecd^1^* flies kept at the restrictive temperature, or following knock down of any of the previously studied ecdysone signaling pathway genes (Fig. 5A). No changes in the number or distribution of developing cysts or of primary spermatocyte clusters were observed (Fig. 5B–F), although detailed counts of each stage could not be made as in the ovary. At the cellular level, we looked for changes in the squamous morphology of the cyst cells similar to those seen in females using electron microscopy. No unwrapping of cysts or conversion to epithelial morphology was observed (Fig. 5G–H, somatic cyst cells pseudocolored green). Therefore, ecdysteroid signaling appears to be critically important for early germ cell development and meiotic entry during female but not in male gametogenesis in Drosophila.

## Discussion

Our studies show that ecdysone signaling promotes multiple, fundamental steps of early oogenesis. Steroid signaling maintains the structure of the GSC niche and allows somatic niche cells to support a normal rather than a reduced number of GSCs. Subsequently, this pathway promotes 16-cell cyst production, meiotic entry and follicle formation. In contrast, male germ cell development lacks a steroid signaling requirement. Despite the fact that male somatic cyst cells interact with developing male germ cells in a very similar manner as in the ovary, and that male cysts form and enter meiosis like their female counterparts, disrupting steroid production or steroid pathway genes for eight days in these cells caused no detectable effect.

Ecdysone signaling was previously reported to be essential for initiating cystoblast development and for cell adhesivity [6]. Germaria from flies in which signaling was reduced using similar methods to those applied here accumulated excess single-spectrosome-containing germ cells (cystoblasts). In contrast, we did not see extra cystoblasts unless knock down flies were followed beyond 8 days. The appearance of extra cystoblasts after prolonged gene knock down correlated with extensive alterations in the normal structure of the GSC niche and anterior germarium. The blockade in cystoblast specification/differentiation is therefore likely to be secondary to changes in somatic support cell shape and function, which are required to limit the range of the BMP signals repressing germ cell differentiation [31];(reviewed in [5]). Consequently, we believe that ecdysone signaling directly affects the processes described here, but is only secondarily involved in cystoblast differentiation.

The formation of 16-cell cysts and entry into meiosis are closely linked. Shortly after completing synchronous mitoses that generate a new 16-cell cyst, all the germ cells enter the first meiosis-specific process, pre-meiotic S phase. The strong reduction in meiotic, 16-cell cyst formation that we observed when ecdysone signaling is reduced, suggests that hormones control meiotic entry during Drosophila oogenesis. Meiosis in many lower organisms is induced by nutrient limitation and modulated by nutrient-sensitive pathways [26]. Ecdysone signals may help determine when cysts have been starved sufficiently to enter meiosis, much as they assess nutrient sufficiency at other decision points.

If steroid signaling in the ovarian soma acts to mediate the extraordinary metabolic demands of female gamete production, then the absence of a male requirement is not surprising. The metabolic demands of egg production are immense, unlike those of sperm production. Thus, decisions affecting oocyte progression may have evolved to employ conserved mechanisms also used during life stage transitions such as dauer formation in *C. elegans* [32] or the larval/pupal transition [33]. This fundamental difference between male and female gametogenesis may apply to a wide range of organisms and might explain why sex-specific steroid signaling is a common aspect of gametogenesis.

Steroid hormone signaling plays a major role in mammalian sex determination and gametogenesis (reviewed in [15], [34]). Transcriptional changes controlled by the Y chromosome-linked SRY gene and hormonal differences dependent on the Sf1 nuclear receptor begin to orchestrate divergent germ cell developmental fates in the bipotential mouse gonad (reviewed in [35]). At this stage, germ cells in both the both male and female gonad are engaged in cyst formation [36]. In females, cysts are completed and enter meiosis while in the testis cyst formation and gamete development arrests. Whether estrogen mediates cyst completion and meiotic entry in female mice in a manner similar to the role of ecdysone in Drosophila remains an interesting question. Squamous, pre-granulosa cells surround mouse germline cysts at the time of follicle formation, and treatment of pregnant animals with estrogen or progesterone enhances the production of multi-oocyte follicles (reviewed in [37]). This raises the possibility that steroid signaling also plays a conserved role during mammalian follicle formation.

## Materials and Methods

### Drosophila Stocks

Experiments were usually conducted on flies 4-–4 days old, raised under standard conditions on yeast/cornmeal/molasses/agar medium. Adult flies were fed yeast paste every third day. c587-GAL4 is described by [38]. The FLP-out strain: *hsFlp; Tub FRT-CD2-FRT-GAL4 UAS-GFP* was a gift from G. Struhl. Lines expressing EcR RNAi (37059), Usp RNAi (16893), and E75 RNAi (44851) were obtained from the Vienna RNAi Stock Center. EcR RNAi was expressed in the presence of UAS-*dcr^2^* to increase the strength of knock down. All other stocks were obtained from the Bloomington Stock Center.

### Genetic Disruption of Ecdysone Signaling

Crosses generating flies of the genotypes c587 GAL4; UAS-USP RNAi/gal80^ts^, c587 GAL4;gal80^ts^;UAS-E75 RNAi, c587;UAS-EcR RNAi/gal80^ts^; UAS-dcr2 and c587 GAL4;UAS-EcR.B1-Δ655.W650A/gal80^ts^ were raised at 20°C to limit RNAi and dominant negative construct expression. Experimental flies were either aged for 8 days at 20^o^C and dissected (0 days at 29°C) or shifted to 29°C within 7 days of eclosion and aged for 4 or 8 days (4 and 8 days at 29^o^C). Gene knock down within a subpopulation of escort cells was achieved using the FLP-out system. Flies expressing hsFlp; Tub FRT-CD2-FRT-GAL4 UAS-GFP (control) and hsFlp; Tub FRT-CD2-FRT-GAL4 UAS-GFP; UAS-USP RNAi, UAS-EcR RNAi or UAS- EcR.B1-Δ655.W650A were raised at 25^o^C, heat shocked in a 37^o^C water bath for 40 minutes and aged for 7 days at 2^o^C. ecd^1^ flies were maintained at 18^o^C. ecd^1^ experimental flies of less than 7 days post-eclosion were either aged for 8 days at 18^o^C and dissected (0 days at 29^o^C) or shifted to 29^o^C and aged for 4 or 8 days (4 and 8 days at 29^ ^C).

### Electron Microscopy

Testes were processed as described [39]. Ovaries were fixed for 1½ hrs in 3% glutaraldehyde, 1% formaldhyde diluted in cacodylate buffer (pH 7.4). The ovaries were then embedded in agarose at 55°C, washed in cacodylate butter and fixed for 1 hour in 1% OsO_4_, 1% KFeCM diluted in cacodylate buffer. Ovaries were then rinsed in water followed by cacodylate buffer and processed as described in [39] from the Maleate rinse.

### Immunofluorescence and Confocal Microscopy

Dissected ovaries and testis were fixed for 10 minutes in 4% paraformaldehyde (Sigma) diluted in Grace’s medium (Lonza Walkersville Inc., USA), and washed three times in PBT (1×PBS, 0.1% Triton-x100, 1 mg/ml BSA). Primary antibodies were diluted in PBT as follows: mouse anti-Hts (1∶20, 1B1, Developmental Studies Hybridoma Bank, USA), mouse anti-FasIII (1∶50, Developmental Studies Hybridoma Bank, USA), guinea pig anti-Tj (1∶1000 [29]), rabbit anti-C(3)G (1∶3000, gift from M. Lilly), mouse anti-USP (1∶200, gift from R. Barrio) and guinea pig anti-Fax (1∶1000, described below). Primary antibodies were incubated overnight at 4°C, washed three times in PBT then incubated overnight at 4°C in secondary antibodies at a dilution of 1∶2000. Secondary antibodies were generated in goat against mouse, guinea pig and rabbit and conjugated with Alexa Fluor 488, 568 and 633 (Invitrogen, USA). Stained tissues were washed twice in PBT and once in PBT with 50 ng/ml DAPI then mounted in Vectashield mounting medium (Vector Labs). Anti-failed axon connections (Fax) was produced by Covance, USA by raising antibodies in guinea pigs against Fax isoform A amino acids 97-294 as described [40]. Confocal images were acquired using a 63x (NA 1.32) PanApo lens and Leica TCS SP5 confocal microscope.

### Analyses of Phenotypic Effects on Ovarian Cells

Ovaries and testis were stained with anti-Hts, anti-FasIII and anti-Tj antibodies. Anti-Hts labels an endoplasmic reticulum-like structure present within germ cells (called a fusome in 2- to 16-cell cysts and a spectrosome in GSCs and CBs) whose number of branches corresponds to cyst size. Anti-Hts staining additionally allows determination of GSC number due to spectrosome positioning relative to cap cells. Follicle formation defects were documented by determining the organization of regions 2a, 2b and 3. Cap cell number was determined by nuclear position and morphology as revealed by anti-Tj staining. To assess ovarian GSC, cyst and cap number, and the frequency of follicle formation defects, 3 independent experiments were performed and a total of 60–100 ovaries were scored. Testis GSC number counts were based on 3 independent experiments and a total of 20–30 testes.

### TUNEL Analysis of Apoptotic Cysts

TUNEL staining was carried out using In Situ Cell Death Detection Kit TMR red (Roche, Germany). Briefly, ovaries were dissected and fixed in 4% paraformaldehyde (Sigma) for 12 minutes. Following fixation, they were washed twice with PBT (PBS/0.1% Triton-x100) and twice with PBS/0.5% Triton-x100. Ovaries were then incubated for 30 minutes at 65°C in 100 mM sodium citrate diluted in PBT followed by three PBT washes. Enzyme diluted 1∶10 in labeling mix was then added and incubated for 2 hours at 37°C. Ovaries were washed in PBT and antibody staining was then performed as described above.

## Supporting Information

Figure S1 **c587 GAL4 drives RNAi mediated knock down of USP in somatic escort and follicle cells.** A–B) c587 GAL4::UASpGFP, UAStGFP 29°C day 7. c587 GAL4 drives strong transgene expression in escort cells (arrowhead) and follicle cells in region 2° (A, magenta arrow), weaker expression is also seen in region 3 follicle cells (A, turquoise arrow). c587 GAL4 does not drive transgene expression germ cells (A, asterisks). A) GFP alone (anti-GFP), B) Green, GFP (anti-GFP), magenta, cell membranes and spectrosome/fusome (anti-Hts and anti-FasIII). C) USP protein is present in the germarium, most highly in escort cells (arrowhead). Lower levels are present in follicle cells in region 2a (magenta arrow) and 3 (turquoise arrow) as well as region 2b (magenta asterisk) and 3 (turquoise asterisk) germ cells. D) After 8 days of c587 GAL4 driving USP RNAi USP protein can no longer be detected within somatic cells of the germarium (arrowhead indicates position of an escort cell nucleus, magenta arrow indicates position of a region 2b follicle cell nucleus and turquoise arrow that of a region 3 follicle cell nucleus). USP expression remains within germ cells of the germaria (region 2b germ cells marked by asterisk, note that there are no region 3 germ cells within this germarium). Scale bar: 10 µm.
(TIF)

Figure S2 **c587 GAL4 drives transgene expression in the somatic cells of the testis.** c587 GAL4::UAS-LacZ 29°C day 7. c587 GAL4 drives transgene expression in somatic cyst cells of the testis (arrow) and cells within the sheath layer (arrowhead). Green: somatic cells (anti-Tj), magenta: fusome (anti-Hts). Scale bar: 10 µm.
(TIF)
