# Supplementary figures and images for: Steroid Signaling within Drosophila Ovarian Epithelial Cells Sex-Specifically Modulates Early Germ Cell Development and Meiotic Entry
Source: PLoS One. 2012 Oct 2;7(10):e46109. doi: 10.1371/journal.pone.0046109 (PMC3462805; doi:10.1371/journal.pone.0046109)

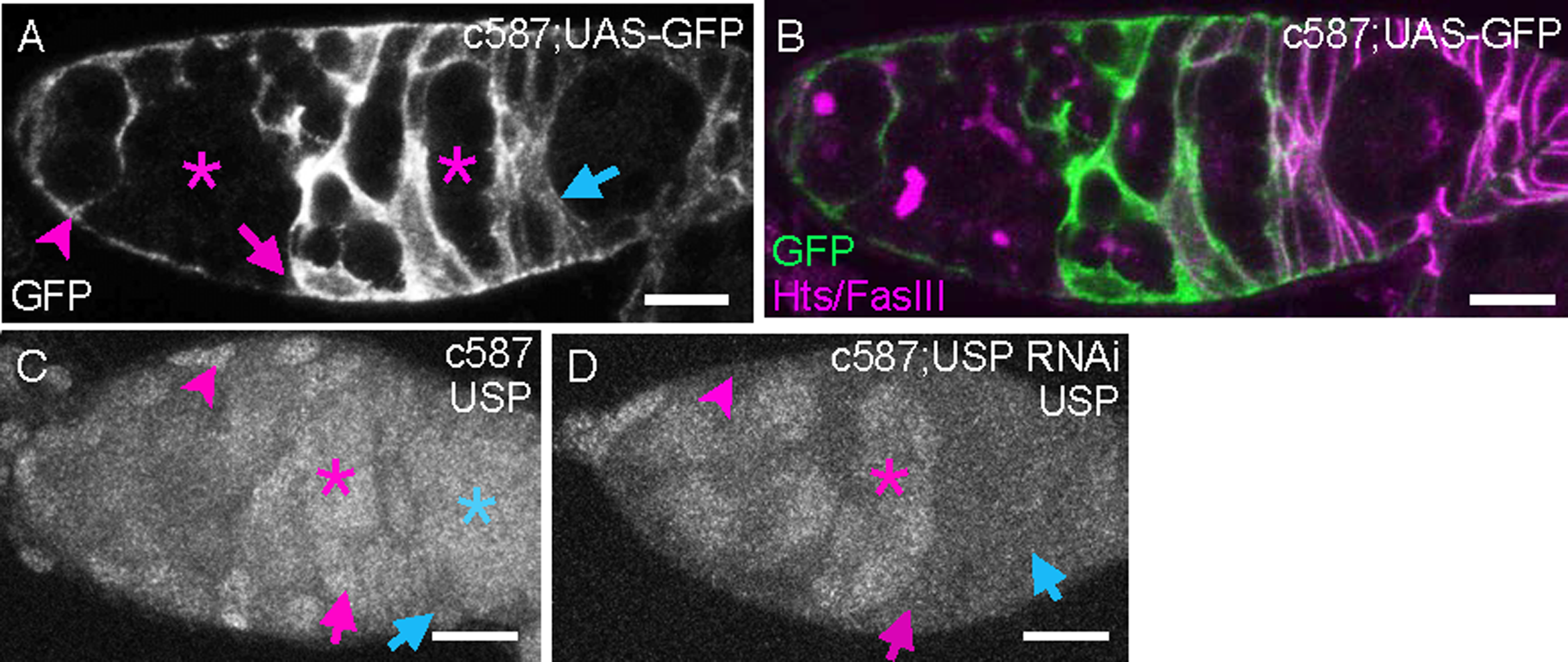

Supplement: Figure S1 — c587 GAL4 drives RNAi mediated knock down of USP in somatic escort and follicle cells. A–B) c587 GAL4::UASpGFP, UAStGFP 29°C day 7. c587 GAL4 drives strong transgene expression in escort cells (arrowhead) and follicle cells in region 2° (A, magenta arrow), weaker expression is also seen in region 3 follicle cells (A, turquoise arrow). c587 GAL4 does not drive transgene expression germ cells (A, asterisks). A) GFP alone (anti-GFP), B) Green, GFP (anti-GFP), magenta, cell membranes and spectrosome/fusome (anti-Hts and anti-FasIII). C) USP protein is present in the germarium, most highly in escort cells (arrowhead). Lower levels are present in follicle cells in region 2a (magenta arrow) and 3 (turquoise arrow) as well as region 2b (magenta asterisk) and 3 (turquoise asterisk) germ cells. D) After 8 days of c587 GAL4 driving USP RNAi USP protein can no longer be detected within somatic cells of the germarium (arrowhead indicates position of an escort cell nucleus, magenta arrow indicates position of a region 2b follicle cell nucleus and turquoise arrow that of a region 3 follicle cell nucleus). USP expression remains within germ cells of the germaria (region 2b germ cells marked by asterisk, note that there are no region 3 germ cells within this germarium). Scale bar: 10 µm. (TIF) [file pone.0046109.s001.tif]

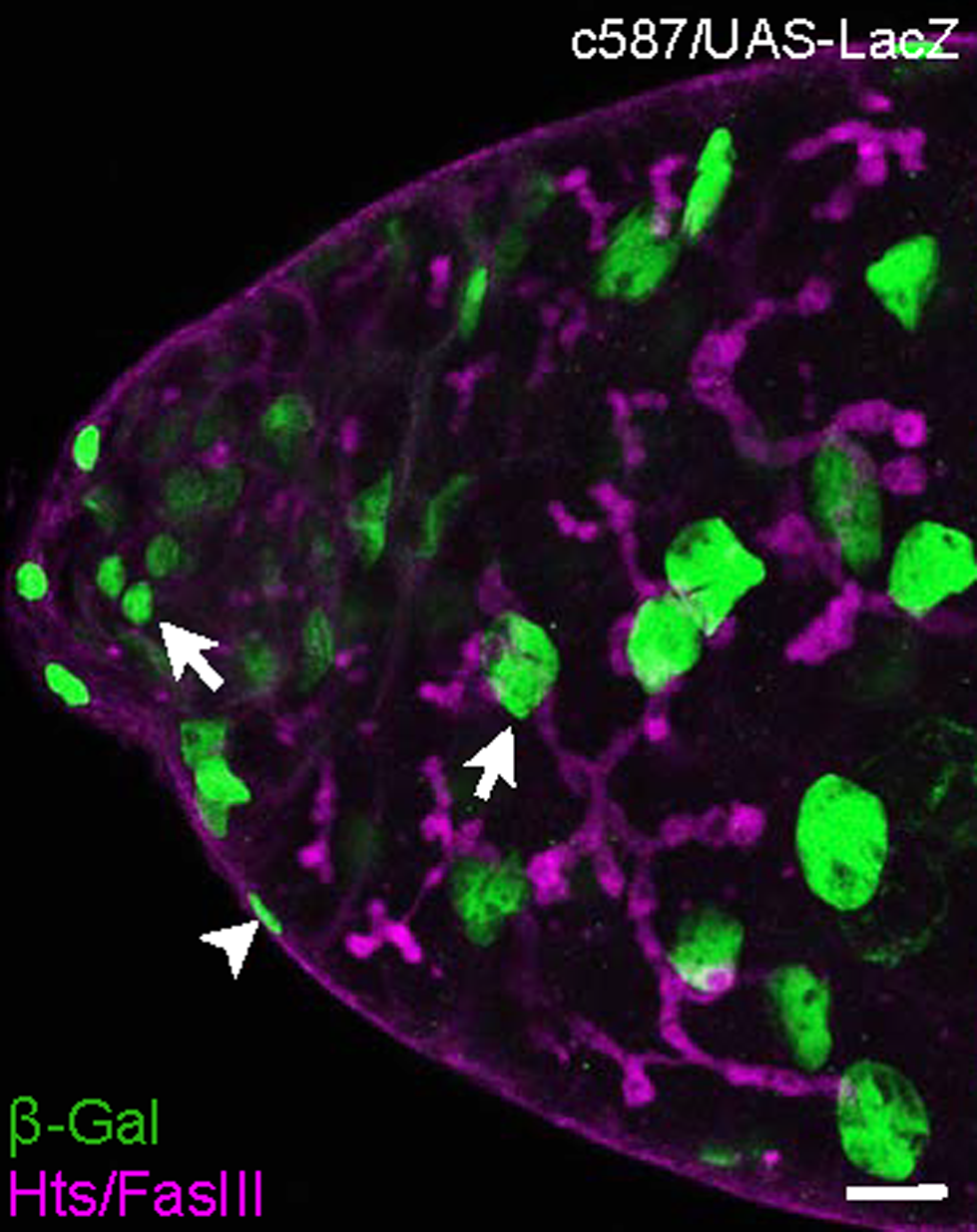

Supplement: Figure S2 — c587 GAL4 drives transgene expression in the somatic cells of the testis. c587 GAL4::UAS-LacZ 29°C day 7. c587 GAL4 drives transgene expression in somatic cyst cells of the testis (arrow) and cells within the sheath layer (arrowhead). Green: somatic cells (anti-Tj), magenta: fusome (anti-Hts). Scale bar: 10 µm. (TIF) [file pone.0046109.s002.tif]
